# The theoretical basis of reservoir pressure in arteries

**DOI:** 10.1113/EP092913

**Published:** 2026-09-30

**Authors:** Kim H. Parker, Alun D. Hughes

**Affiliations:** ^1^ Department of Bioengineering Imperial College London UK; ^2^ Institute of Cardiovascular Science University College London London UK

**Keywords:** blood pressure, haemodynamics, reservoir‐excess pressure analysis, two‐time analysis

## Abstract

The separation of measured arterial pressure into a reservoir pressure and an excess pressure was introduced over 20 years ago as a heuristic hypothesis. Since then it has gained some traction through epidemiological studies that show that various measures of reservoir and excess pressure are independent risk factors for cardiovascular disease. We demonstrate that a two‐time asymptotic analysis of the 1‐D conservation equations in each artery coupled with the separation of the arteries into inviscid and viscid arteries, based on their resistance or drag coefficients, results in a formal derivation of the reservoir pressure. The most important result of this analysis is that flow in the arteries can be considered quasi‐steady during every interval on the slow time scale. The slow‐time conservation equations involve a drag coefficient that models the effect of viscosity on the flow. We separate the arteries into the larger inviscid arteries where viscosity is negligible and the smaller viscid arteries where it is not. The slow time pressure in the inviscid arteries is shown to be spatially uniform but varying in time. We identify this pressure as the reservoir pressure. Dynamic analysis using mass and energy conservation in the inviscid arteries shows that the reservoir pressure accounts for the storage of potential energy by the distension of the elastic inviscid arteries during early systole and its release during late systole and diastole. This analysis thus provides a formal derivation of the reservoir pressure and ascribes its physical meaning.

## Abstract (highlights)

- **What is the central question of this study?**
  Can reservoir pressure be derived from the first principles of fluid mechanics?
- **What is the main finding and its importance?**
  Reservoir pressure represents the recoverable work due to the deformation of the elastic inviscid arteries. These are defined as the larger arteries in which viscosity effects are negligible. Excess pressure, defined as the difference between the measured pressure and the reservoir pressure, represents local energy losses over the cardiac cycle. This separation may lead to a better physical understanding of haemodynamics.

## 1 INTRODUCTION

The most important difference between blood flow in the arteries and the more familiar fluid flow in pipes and ducts is that the arteries are deformable. The pressure that drives the flow also deforms the arteries in which the blood is flowing and this must be taken into account when analysing the flow. The effect is particularly important when the flow is unsteady; and arterial flow is intrinsically unsteady due to its pulsatile nature.

The blood pressure and flow waveforms in the circulation are morphologically complex, differ from one another, and both change with location in the circulation (Caro et al., 2012). These features result from the complex nature of wave transmission and reflection phenomena in the highly branched circulation (Segers et al., 2017). This complex behaviour has led to a range of simplified models being used to understand circulatory behaviour. The two‐element Windkessel model of arterial haemodynamics is well known, but despite extensive efforts by Otto Frank and his followers, it does not describe arterial pressure during systole with enough precision to be clinically useful. Consequently, it is generally seen by the cardiovascular community as largely of historical or didactic interest (Westerhof et al., 2019).

During diastole, however, the Windkessel model provides an excellent description of arterial pressure, particularly in the elderly; the time constant of the observed exponential fall‐off of pressure, RC, where R is the net arterial resistance and C is the net compliance of the arteries, is measured clinically, particularly in the pulmonary circulation (Hadinnapola et al., 2015; Lankhaar et al., 2008), and is frequently cited as one of the descriptors of the performance of the cardiovascular system. More complex three‐ or four‐element Windkessel models have been developed, but while more elements provide better fits, the parameter estimates of these models may be inaccurate (Lambermont et al., 1997; Segers et al., 2008), and for models with four or more elements, the physiological meaning of the additional elements can be obscure (Burattini & Gnudi, 1982; Segers et al., 2008).

Reservoir pressure was introduced to arterial haemodynamics as part of a heuristic hypothesis, subsequently termed the reservoir‐wave hypothesis (Wang et al., 2003). In that paper, the reservoir pressure was called the Windkessel pressure and was calculated from locally measured pressure and velocity using the classical two‐element Windkessel theory (Frank, 1990).

In a follow‐up paper, the local ‘Windkessel pressure’ was observed to propagate along the aorta at the local wave speed, unlike pressure in Windkessel theory, which is assumed to be uniform throughout the arteries. This component of pressure was renamed the reservoir pressure to denote this difference, and the hypothesis was reformulated to account for the observed propagation of the reservoir pressure down the aorta (Tyberg et al., 2009). To clarify that both reservoir and excess pressure arise from waves, the hypothesis was renamed the reservoir‐excess pressure hypothesis (Hughes & Parker et al., 2020) and we use this terminology subsequently.

The reservoir‐excess hypothesis in its most general form asserts that it may be useful to separate arterial pressure into two components: the reservoir pressure and the excess pressure. The reservoir pressure waveform is assumed to be the same in every artery but delayed by the wave travel time from the aortic root. The excess pressure is defined as the difference between the measured pressure and the reservoir pressure and varies both in time and space (Figure 1).

**FIGURE 1:**
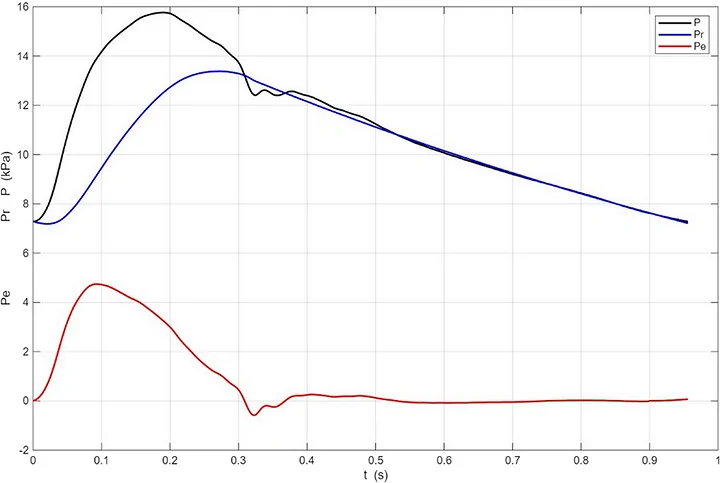
The pressure *P* in the brachial artery (black line) calculated by a 1‐D computational model (Willemet & Alastruey, 2015). The reservoir pressure $P_{r}$ (blue line) is calculated using a method that assumes the flow is proportional to the external pressure (Parker, 2017). The excess pressure $P_{e}$ (red line) is the difference between P and $P_{r}$.

Mathematically this can be expressed

(1) $$P\left(x,t\right)=P_{r}\left(t-\delta t\left(x\right)\right)+P_{e}\left(x,t\right)$$

where P is the measured pressure, $P_{r}$ is the reservoir pressure, $P_{e}$ is the excess pressure, and x and t are the spatial and temporal variables, respectively. δt is a time delay that depends on the distance from the aortic root. An analysis using the calculus of variations showed that the reservoir pressure, so defined, required the minimum hydraulic work for any given flow waveform (Parker et al., 2012).

The reservoir‐excess pressure hypothesis is an attempt to retain the ability of the Windkessel model to describe diastolic haemodynamics while positing the excess pressure to explain the difference between measured arterial pressure and the reservoir pressure during systole. When the hypothesis was first formulated, we were unable to provide theoretical grounds for the existence of the reservoir pressure and were careful to describe it as a heuristic hypothesis.

The concept of a reservoir pressure elicited some controversy amongst haemodynamicists (Mynard & Smolich, 2014; Westerhof et al., 2015). Nevertheless, a number of studies have shown the utility of the concept (see for example, Aizawa et al., 2021; Behnam et al., 2019; Hametner et al., 2014; Narayan et al., 2015). The studies are too diverse to summarise in detail but generally it has been found that parameters associated with the reservoir pressure, such as its peak value or area under the curves of reservoir or excess pressure are associated with future risk of cardiovascular disease, independent of other risk factors.

Encouraged by these findings, we explore the theoretical and physical basis of the reservoir and excess pressures.

## 2 THEORETICAL BACKGROUND

This analysis is based on the two fundamental principles of continuum fluid mechanics: the conservation of mass and momentum. The conservation of mass is one of the basic tenets of non‐relativistic physics which, coupled with the near incompressibility of blood, can be expressed as a conservation of the volume of blood:

(2) $$\frac{dV}{dt}=Q_{\mathrm{in}}\;-Q_{\mathrm{out}}$$

where V is the control volume, $Q_{\mathrm{in}}$ and $Q_{\mathrm{out}}$ are the volume flow rates into and out of the control volume. If we model individual arteries as uniform compliant tubes, and apply mass conservation to a differential element of the artery of length dx, where x is the axial distance, Equation (2) can be written in each artery as the partial differential equation:

$$A_{t}+UA_{x}+AU_{x}=0$$

where A is the cross‐sectional area, U is the average velocity so that Q=UA and we have used subscript notation to indicate partial derivatives of the independent variables x and t.

The conservation of momentum is the application of Newton's second law to the control volume that is central to the Eulerian analysis of fluid mechanics. Applying it to the differential element of an artery:

$$\rho \left(U_{t}+UU_{x}\right)=-P_{x}-\sigma$$

where ρ is the density of blood, P is the pressure and σ is the wall shear stress due to viscosity. Writing this equation in a similar format, we obtain the 1‐D conservation equations for flow in an artery:

(3) $$\begin{array}{c} A_{t}+UA_{x}+AU_{x}=0\\ U_{t}+\frac{1}{\rho }P_{x}+UU_{x}=-RU \end{array}$$

where we have written σ=RU where R is the drag coefficient. These two equations contain three dependent variables, and it is necessary to provide another equation, usually called the *tube law* that describes the relationship between P and A for the compliant artery. We make the simplest assumption that each artery is spatially uniform and that the tube law has the functional form *A* = *A*(*P*). Detailed anatomical measurements of individual arteries show that they are slightly tapered (Caro et al., 2012). Experimental measurements of wave travel in tapered deformable tubes show that small taper has a small effect on wave dynamics (Gerrard, 1985), which suggests that our model of the arteries as a binary tree of uniform deformable tubes is adequate for our approximate asymptotic analysis.

The next step in this analysis is critical but based upon some very subtle and deep mathematical analysis that is difficult to describe rigorously and succinctly (Parker, 2009). Briefly, this system of non‐linear partial differential equations is hyperbolic in form and can be solved by the method of characteristics. (John von Neumann, one of the most eminent mathematicians of the late 20th century, replied to a physicist colleague who said ‘I'm afraid I don't understand the method of characteristics’: ‘Young man. In mathematics you don't understand things. You just get used to them.’ [WikiQuotes]) This solution has the form of a wave that propagates with the wave speed $c={\left(\frac{A}{\rho }\frac{dP}{dA}\right)}^{\frac{1}{2}}$. Using this relationship and the tube law, we can eliminate A from the conservation equations:

(4) $$\begin{array}{c} P_{t}+UP_{x}+\rho c^{2}U_{x}=0\\ U_{t}+\frac{1}{\rho }P_{x}+UU_{x}=-RU \end{array}$$

For Poiseuille flow, $R=\frac{8\pi \mu }{\rho A}$, where μ is the coefficient of viscosity. These 1‐D equations are based on a number of approximations which have been discussed in detail previously and are the basis of wave intensity analysis which describes arterial haemodynamics in terms of forward and backward travelling wavelets (Parker, 2009).

### 2.1 Two‐time asymptotic analysis

In this section we introduce a new analysis based on the two‐time asymptotic method to justify the separation of P into two components. The analysis takes advantage of the large difference between the wave speed c and the convective velocity U which is usually expressed in terms of the Mach number, $m=\frac{U}{c}\;\ll 1$.

It is essential in asymptotic analysis to deal with non‐dimensional equations since the magnitude of a dimensional variable depends upon the units used to describe it. The choice of characteristic values to non‐dimensionalise the dimensional variables is generally straightforward; for example, the characteristic length $L^{*}$, characteristic velocity $U^{*}$, characteristic time $\frac{L^{*}}{U^{*}}$, and characteristic wave speed $c^{*}$. The choice of the characteristic pressure $P^{*}=\rho c^{*}U^{*}$, however, is not immediately obvious and warrants some comment. Experimenting with other characteristic pressures shows that this is the only choice that does not result in secular terms, asymptotically large terms that lead to divergence of the perturbation equation. Using these characteristic values, we obtain the non‐dimensional form of the conservation equations:

(5) $$\begin{array}{c} p_{t}+up_{x}+\frac{1}{m}u_{x}=0\\ u_{t}+\frac{1}{m}p_{x}+uu_{x}=-ru \end{array}$$

where $p=\frac{P}{P^{*}}$, $u=\frac{U}{U^{*}}$ and $r=\frac{L^{*}R}{U^{*}}$ is the non‐dimensional drag coefficient. For convenience we have retained the same symbols for dimensional and non‐dimensional distance and time.

These equations describe the flow in the individual arteries. To extend the analysis to the arterial tree, we have to derive the conservation conditions at the bifurcations connecting the individual arteries. Assuming that the bifurcations are discontinuities, the matching conditions can be written:

(6) $$q_{a}=q_{b}+q_{c}$$

(7) $$p_{a}=p_{b}=p_{c}$$

where q is the non‐dimensional volume flow rate, a is the index of the parent artery, and b and c are the indices of the daughter arteries. These conditions are generally known as the *Kirchoff conditions* in network theory.

In the two‐time analysis we assume that there are two distinct times; a slow time T=t commensurate with convection and a fast time $\tau =\frac{1}{m}\;t$ commensurate with wave travel times. By the chain rule for partial derivatives, the partial derivative with respect to t can be written

$$\;\frac{\partial }{\partial t}=\frac{\partial }{\partial T}+\frac{1}{m}\frac{\partial }{\partial \tau }$$

We also assume that the dependent variables p and u can be expanded as regular asymptotic series in the slow time scale:

$$\begin{array}{c} p\left(x,T,\tau \right)=p_{0}\left(x,T,\tau \right)+mp_{1}\left(x,T\right)+m^{2}p_{2}\left(x,T\right)+O\left(m^{2}\right)\\ u\left(x,T,\tau \right)=u_{0}\left(x,T,\tau \right)+mu_{1}\left(x,T\right)+m^{2}u_{2}\left(x,T\right)+O\left(m^{2}\right) \end{array}$$

Substituting these expansions into the conservation equations and collecting orders of m, we obtain the following set of equations at different orders:

$O(\frac{1}{m})$

(8) $$\begin{array}{c} p_{0\tau }+u_{0x}=0\\ u_{0\tau }+p_{0x}=0 \end{array}$$

$O\left(1\right)$

(9) $$\begin{array}{c} u_{1x}=-\left(p_{0T}+u_{0}p_{0x}\right)\\ p_{1x}=-ru_{0}-\left(u_{0T}+u_{0}u_{0x}\right) \end{array}$$

$O\left(m\right)$

(10) $$\begin{array}{c} u_{2x}=-u_{1}p_{0x}-\left(p_{1T}+u_{0}p_{1x}\right)\\ p_{2x}=-ru_{1}-u_{1}u_{0x}-\left(u_{1T}+u_{0}u_{1x}\right) \end{array}$$

The solution of these equations using the appropriate boundary conditions describes the flow in each of the uniform arteries that make up the arterial tree.

### 2.2 Relating results in uniform individual arteries to the arterial tree

The solution of differential equations depends not only on the equations but also on the boundary and initial conditions. We have ignored this problem in the previous section by looking only at the generic nature of the solutions in a uniform elastic tube without any reference to the boundary conditions. Applying appropriate boundary conditions to arteries is difficult because of the anatomical complexity of the arterial system. The arterial system can best be described as a binary tree structure with occasional loops (e.g. the circle of Willis in the cerebral circulation, the palmar and plantar arches in the hand and foot, and the highly looped arterial arcades supplying the intestines). The total number of arteries in the circulation depends on the definition of *terminal arterioles*, the boundary between the arterial network and the capillary microcirculation, but it is certainly a very large number.

The total number of capillaries in the human body can be estimated in two ways: by the weighted average of measured density of capillaries in various tissues or by average capillary flow rates compared to cardiac output. Both methods give roughly the same estimate, 10 billion capillaries ($10\times 10^{9}$) in man. Making the anatomically conservative estimate that each arteriole perfuses at least 10 capillaries (Delashaw & Duling, 1988), there are roughly $10^{9}$ terminal arterioles. In a binary tree with *N* terminal branches there are *N* − 1 supporting branches, which means that the total number of arteries is estimated to be $2\times 10^{9}-1$. In an arterial tree with loops, the estimate is greater because the arteries in the loops do not directly support terminal branches.

Two billion arteries preclude any exact modelling of the arterial system with current computer technology. There are two viable alternatives: statistical methods and integrative methods. Statistical methods are attractive and worth exploring but they rely on extensive measurements and modelling to produce clinically useful results. We will use an integrative approach in this paper.

The conservation of mass, particularly for incompressible fluids, is most amenable to the integrative approach because the rate of change of a control volume is directly related to the net flux into the volume. We can exploit this to study the dynamics of the whole arterial volume.

### 2.3 Fast time solution

The conservation equations to $O(\frac{1}{m})$ are given in Equation (8). Cross differentiation and subtraction gives the canonical wave equation (the non‐dimensional wave speed has been normalised to 1):

(11) $$p_{\tau \tau }=p_{xx}\;\;\mathrm{or}\;\;u_{\tau \tau }=u_{xx}\;$$

Converting back to dimensional variables gives the dimensional wave equations:

(12) $$P_{tt}=c^{2}P_{xx}\;\mathrm{or}\;\;U_{tt}=c^{2}U_{xx}$$

The solution of the wave equation that satisfies the boundary conditions can be expressed as a combination of basis waves. Historically, the most popular method is Fourier analysis based on sinusoidal waves (Westerhof et al., 1969). We will take an alternative approach, originally developed to study waves in gas dynamics, that uses discrete wavefronts (wavelets) as the basis of the analysis (Courant & Hilbert, 1962). This approach has a number of advantages: any waveform, including periodic waveforms, can be synthesised using successive wavelets of the appropriate magnitude, and it is conceptually easier to determine the reflection and transmission of the waves when they encounter the discontinuities at nodes.

It is easy to see that any function f(z) is a solution of the wave equation when z=x±ct because by the chain rule for partial differentiation $f_{xx}=f^{\prime \prime }\;\left(z\right)$ and $f_{tt}=c^{2}\;f^{\prime \prime }\left(z\right)$ where 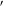 denotes the total derivative. For a wavelet, we chose the step function f=ΔPU where ΔP is the amplitude and U is the unit step function; ΔP can be either positive or negative. When z=x−ct the wavelet is travelling in the forward direction and when z=x+ct, the backward direction. Making the Galilean transformation to travel with the wavelet, conservation of mass and momentum show that when the effect of viscosity is negligible the magnitude of the change in pressure across the wavelet is related to the magnitude of the change in velocity:

(13) ΔP=±ρcΔU

where plus is for forward waves and minus, backward. This equation is generally referred to as the *water hammer* (or Joukowsky) equation.

Wavelets are generated whenever local conditions are not in equilibrium, and they propagate at the local wave speed in order to reestablish local equilibrium. Each wavelet carries information about the changes in conditions where it was generated. Whenever a wavelet encounters a node, an internal bifurcation or an external terminus, it will experience new conditions. If these conditions are not in equilibrium with the local conditions, new wavelets will be generated so that local equilibrium is established. The wavelets will proliferate and propagate throughout the arterial tree until a new equilibrium is established throughout the tree.

Calculating arterial dynamics by tracking wavelets is not practical because of the size of the arterial tree and the exponential growth of the number of wavelets as they propagate information about local equilibrium throughout the tree (Parker, 2017). It does, however, provide an excellent framework for understanding the results of the two‐time asymptotic method. The time it takes to establish new equilibrium conditions depends on the wave speed of the wavelets. As the Mach number increases the propagation time gets shorter compared to the convection times that establish the boundary conditions at the external nodes: the inlet to the aorta and the terminal arterioles that perfuse the microcirculation. As m→0, the wavelets establish equilibrium conditions that satisfy the instantaneous boundary conditions; the *quasi‐steady* solution.

The steady‐state solution in a rigid tree of uniform branches is conceptually straightforward (Kakuba et al., 2021). When the vessels are elastic, the solution is computationally more difficult because the area of the branches, and hence their resistance, depends upon the local pressure, but it is still straightforward in principle. The solution algorithm starts at the outlets of the terminal arteries where the resistance due to the microcirculation is known (or assumed). The inlet resistance of the terminal arteries is the sum of the outlet resistance and the resistance of the artery. At each bifurcation the outlet resistance of the parent vessel is determined by the inlet resistances of the daughters acting in parallel. The inlet resistance of the parent is the sum of its outlet resistance and its own resistance. Iterating this calculation through all of the branches yields the inlet resistance of the root artery. Given the inlet resistance at the root $R_{0}$, the mean pressure and volumetric flow rate at the root can be determined using $P_{0}\left(T\right)=Q_{0}\left(T\right)R_{0}\left(T\right)$.

The solution algorithm continues by working forward through the tree. The pressure drop through any artery is $\Delta P_{n}=Q_{n}R_{n}$. Applying this to the root artery gives the pressure at the outlet at the first bifurcation, which, by the Kirchoff conditions, is equal to the pressure at the inlet of the two daughter arteries. The net flow into each bifurcation is zero, which means that, for a bifurcation with parent artery a and daughter arteries b and c, $Q_{a}=Q_{b}+Q_{c}$ and the distribution of flow between the daughter arteries depend on their inlet resistances $\frac{Q_{b}}{Q_{c}}=\frac{R_{c}}{R_{b}}$. Iterating this process down to the terminal arteries gives the quasi‐steady solution for the flow and pressure throughout the arterial tree at each interval of the slow time T.

Practically, this quasi‐steady solution is only conceptual, simply because of the size of the arterial tree; exact knowledge of the anatomy and physical properties of 2 billion arteries is unobtainable. Advances in our understanding of arterial haemodynamics in the foreseeable future must rely on intelligent physiological modelling.

### 2.4 Slow time solution

The O(1) Equations (9) give the O(1) variables as functions of the $O(\frac{1}{m})$ variables:

(14) $$\begin{array}{c} u_{1x}=-\left(p_{0T}+u_{0}p_{0x}\right)\\ p_{1x}=-ru_{0}-\left(u_{0T}+u_{0}u_{0x}\right) \end{array}$$

The terms in brackets on the right‐hand side of the equations are the convective derivative of the two variables, $p_{0}$ and $u_{0}$; that is, the rate of change of the variable following an element of fluid. They are both equal to zero because $p_{0}$ and $u_{0}$ are constant in uniform arteries. The first order variables on the slow time scale are thus:

(15) $$\begin{array}{c} u_{1x}=0\\ p_{1x}=-ru_{0} \end{array}$$

The absence of T in these equations means that the solutions at this order are also quasi‐steady. Converting back to dimensional variables, the solution in each artery at each time T on the slow time scale is

(16) $$\begin{array}{c} U_{1}\left(x\right)=\mathrm{constant}\\ P_{1}\left(x\right)=P_{1}\left(0\right)-RU_{1}x \end{array}$$

where x=0 is the inlet of the artery. The quasi‐steady solution for the arterial tree can be found by an iterative algorithm starting at the terminal arteriole that calculates the resistances of each path through the tree. The flow and the pressure drop through each artery can then be calculated by working forward through the tree (Kakuba et al., 2021). Using this method the instantaneous quasi‐steady pressure and flow throughout the arterial tree can be calculated.

This solution procedure is conceptually simple but impractical because of the large number of arteries and the need to know their anatomical and structural properties. We note, however, that a simple solution is possible if the resistance coefficient R is negligible. This is the motivation for the next stage of the analysis.

The O(m) Equations (10) give the O(m) variables as functions of the lower order solutions:

(17) $$\begin{array}{c} u_{2x}=-u_{1}p_{0x}-\left(p_{1T}+u_{0}p_{1x}\right)\\ p_{2x}=-ru_{1}-u_{1}u_{0x}-\left(u_{1T}+u_{0}u_{1x}\right) \end{array}$$

At this order the convective derivative terms are no longer zero, and the non‐linear interaction between velocity, area and pressure begin to affect the asymptotic analysis. Using the solution at the lower orders, the equations for the second order terms are:

(18) $$\begin{array}{c} u_{2x}=ru_{0}^{2}\\ p_{2x}=-ru_{1} \end{array}$$

The physical interpretation of these equations is that the velocity is increasing along the artery because the fall in pressure due to viscous effects causes a decrease in the area of the artery, while the pressure is falling further because of the additional velocity at the first order. These extra terms increase the complexity of the steady‐state solution for the arterial tree but do not affect the solution algorithm.

It must be remembered that the equations presented in this section are for the flow in individual, uniform arteries and the steady‐state solutions we refer to are the quasi‐steady solutions for the entire arterial tree that depend on the boundary conditions that are changing on the slow time scale. These solutions exist in the sense that there is an algorithm for finding them (Kakuba et al., 2021). Practical solutions that could be used clinically, however, would require God‐like knowledge of the anatomy and properties of the billions of arteries that make up the arterial tree. There are, however, approximate asymptotic solutions that may be useful. These are discussed in the next section.

## 3 INVISCID AND VISCID ARTERIES

We make the novel and important assumption that the arteries can be divided into *inviscid* arteries where viscous effects are negligible and *viscid* arteries where they are not negligible. Viscous effects are included in our 1‐D conservation equations for flow in individual arteries through the drag coefficient R, which assumes that the drag due to wall shear is a linear function of the mean velocity U. This assumption is simplistic, particularly under highly unsteady conditions, but is useful in our asymptotic analysis of the problem. In that spirit, we further assume that the drag coefficient is given by the Poiseuille flow drag $R=\frac{8\pi \mu }{\rho A}$. The non‐dimensional drag coefficient $r=\frac{8\pi \mu L^{*}}{\rho AU^{*}}$, which can be used to quantify what is meant by ’negligible’.

To motivate the discussion, we note that boundary layer theory, probably the most successful approximation in fluid mechanics, is based on the non‐dimensional Reynolds number Re=ρU∗D∗μ where $U^{*}$ and $D^{*}$ are the characteristic velocity and diameter. It is the ratio of inertial to viscous forces in the flow and is used to separate regions of flow into inviscid regions where viscosity is negligible and boundary regions where it is not. Many problems can be solved by solving separately in the inviscid and boundary regions and matching the two solutions at the boundary of the boundary layer.

In our analysis of flow through individual arteries, we have taken the characteristic length to be the length of the artery $L^{*}$. Writing $A=\frac{\pi }{4}D^{*2}$, we can write the non‐dimensional viscous resistance parameter, r=32ReDL*D*, where $Re_{D}$ is the Reynolds number based on the diameter. Thus, when the Reynolds number is large, r≪1 and we can assume that flow is approximately inviscid.

### 3.1 Inviscid arteries and the reservoir pressure

From the results of the previous section, we see that as r→0, both P and U are constant along the length of each artery. By the Kirchoff conditions, the pressure at the outlet of a parent artery is equal to the pressures at the inlets of the two daughter arteries. If r is also negligible in the daughter arteries, the quasi‐steady pressure will be uniform in all three arteries. Extending the argument to successive bifurcations, it is possible to define an inviscid region made up of contiguous arteries which can be considered to be inviscid to O(r), in which P is uniform. It is not constant, however, because the boundary conditions that determine the quasi‐steady P are changing on the slow time scale, making P=P(T).

The original heuristic reservoir‐excess hypothesis conjectured the utility of separating the measured arterial pressure P(x,t) into two components: a reservoir pressure $P_{r}\left(t\right)$ that depends on the net compliance of the arterial system and an excess pressure $P_{e}\left(x,t\right)=P-P_{r}$. As a spatially averaged property, $P_{r}$ depends only on time $P_{r}\left(t\right)$ while $P_{e}$ depends on both position in the arterial tree and time. From the above analysis, we see that the pressure in the inviscid arteries satisfies the definition of the reservoir pressure on the slow timescale t=T, thus providing a theoretical derivation of $P_{r}$.

The separation of arteries is illustrated in Figure 2, where the larger inviscid arteries are coloured green and the smaller viscid arteries are coloured red. This figure should be considered as a cartoon that depicts how the inviscid arteries can be widely distributed while having similar properties, rather than being anatomically correct.

**FIGURE 2:**
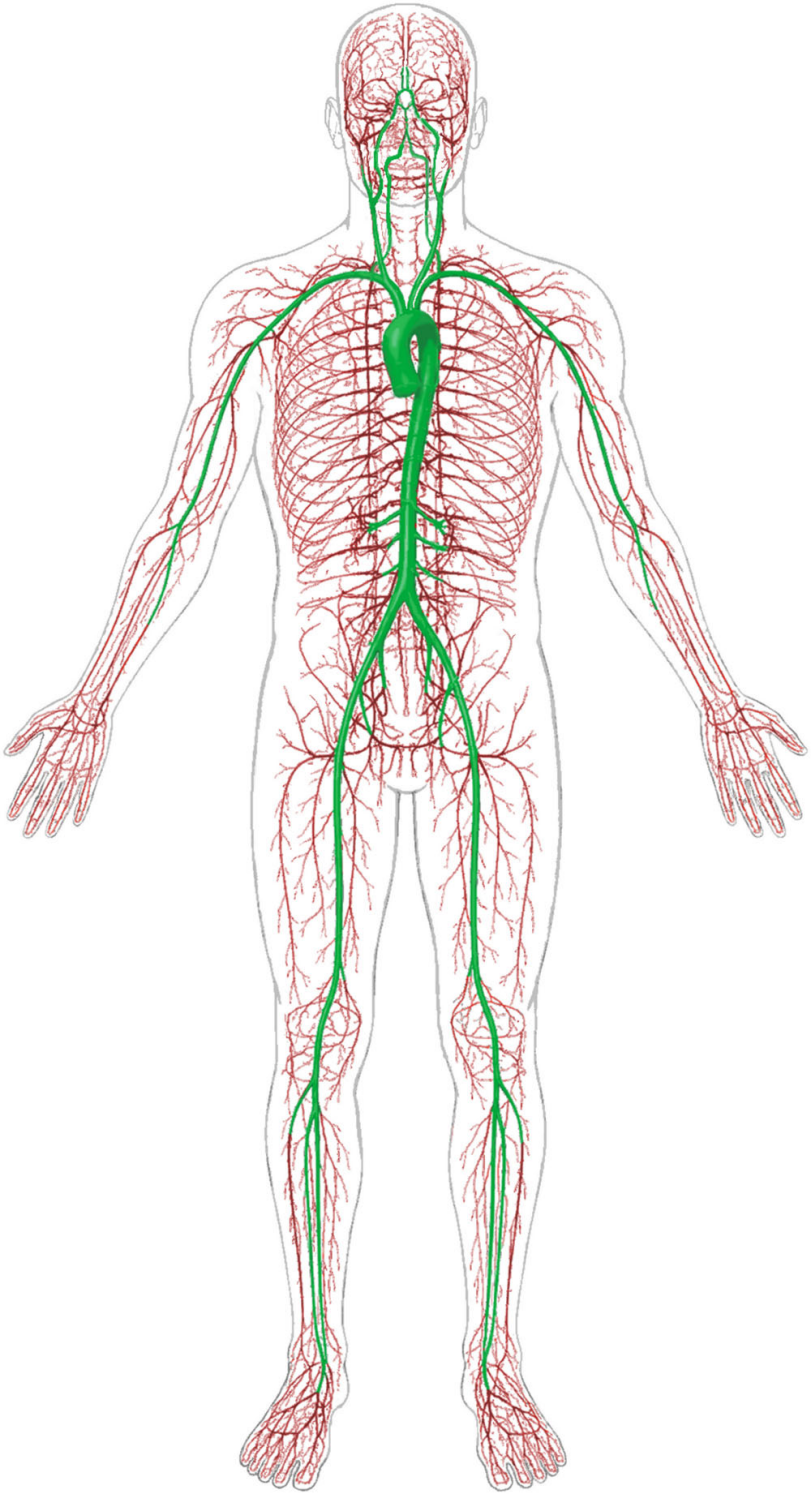
A sketch of the inviscid (green) and viscid (red) arteries. The larger, inviscid arteries are loosely based on data from the ADAN project (Blanco et al., 2015). The smaller, viscid arteries are not anatomically correct but are generated to illustrate the space‐filling nature of the small arteries.

Since $P_{r}\left(t\right)$ is uniform in the inviscid arteries it is easiest to analyse its dynamics using a compartmental analysis based on a control volume V defined as the lumen of the largest set of contiguous arteries with r less than a chosen threshold. The inlet is the aortic root, and the outlets are the outlets of the set of terminal inviscid arteries K. Conservation of incompressible blood Equation (2) for this control volume can be written in terms of $P_{r}$ if we assume that V is related to $P_{r}$ through the compliance $C=\frac{dV}{dP_{r}}$, which we assume to be constant over the range of pressures during the cardiac cycle. $Q_{\mathrm{in}}=\frac{P_{0}}{R}$ where $P_{0}=P_{r}+P_{e}$ and R is the inlet resistance at the root of the aorta. We also assume that the venous pressure is negligible and that the resistance to flow through the viscid arteries and the microcirculation downstream from each terminal artery $R_{k}$ is constant so that $Q_{k}=\frac{P_{r}}{R_{k}}$. Using these assumptions:

(19) $$C\;\frac{dP_{r}}{dt}=\frac{P_{r}+P_{e}}{R}\;-\sum_{k\in K}\frac{P_{r}}{R_{k}}$$

Since the outlet resistances are in parallel, we can write $\frac{1}{R}=\sum_{k\in K}\frac{1}{R_{k}}$. Substituting, the conservation equation reduces to the ordinary differential equation:

(20) $$\mathrm{RC}\;\frac{dP_{r}}{dt}=P_{e}\left(t\right)$$

We recognise RC as the time constant for the inviscid arteries, which is frequently called τ but we have used that symbol to denote the fast time scale and will retain RC in our analysis.

### 3.2 The physical meaning of reservoir pressure

Like mass and momentum, energy must be conserved in a control volume. Energy conservation, however, is not straightforward because energy can take many forms. For example, viscous effects in fluids, like friction in solid body interactions, can convert mechanical energy into heat. This form of energy conversion is very difficult to measure directly and in most cases is deduced by assuming that energy is conserved. Energy conservation is, however, useful in understanding the physical nature of the reservoir pressure.

Consider the energy balance in the control volume V defined by the inviscid arteries, which are assumed to be uniform and elastic (i.e. their deformation is reversible). Since the cardiovascular system is effectively isothermal, the conservation of energy for the elastic inviscid arteries is given by the balance between energy and the work done:

(21) $$\int_{V}\frac{\partial e}{\partial t}dV=W_{\mathrm{in}}\;-W_{\mathrm{out}}-W_{\mathrm{elas}}$$

where $e=P+\frac{1}{2}\rho U^{2}$ is the energy density of a fluid element, $W_{\mathrm{in}}=PQ$ is the hydraulic work at the inlet of the aorta, $W_{\mathrm{out}}=\sum_{k\in K}PQ_{k}$ at the outlet of the terminal inviscid arteries K and $W_{\mathrm{elas}}=P\frac{dV}{dt}$ is the elastic work done on the elastic walls of the inviscid arteries. From the previous section, the pressure in the control volume is $P_{r}$ and the pressure at the inlet of the aorta is $P_{r}+P_{e}$. Since $P_{r}$ is uniform within the inviscid arteries, we can evaluate the integral and substitute the relevant pressures:

$$\frac{\rho }{2}\int_{V}\frac{U^{2}}{dt}dV=\left(P_{r}+P_{e}\right)Q_{0}+P_{r}\sum_{k\in K}Q_{k}+P_{r}\frac{dV}{dt}$$

Using the results from the previous section, conservation of mass gives $\frac{dV}{dt}=Q_{0}\;-\sum_{k\in K}Q_{k}$. Substituting, the terms involving $P_{r}$ vanish giving:

(22) $$P_{e}Q_{0}=\frac{\rho }{2}\;\int_{V}\frac{U^{2}}{dt}dV$$

We see from this result that the reservoir pressure $P_{r}$ is the fraction of the measured pressure that accounts for the work done on the elastic arteries, while the excess pressure $P_{e}\left(x,t\right)$ accounts for the net changes in the kinetic energy of blood within the inviscid arteries.

The elastic energy of the walls of large elastic arteries is negligibly dissipative (Caro et al., 2012), so any increase in elastic energy during early systole will be almost completely recovered during late systole and diastole. This is the Windkessel effect, which helps to buffer the highly pulsatile pressure developed by the ventricle. The changes in kinetic energy, however, are not necessarily symmetrical, for example surging between regions with different compliances, and can result in losses during the cardiac cycle (Parker et al., 2012).

### 3.3 Physiological and clinical use of reservoir and excess pressure

We cannot be certain how reservoir and excess pressure will be used in the future; to date they have mainly been used in clinical or epidemiological studies which show that various parameters associated with them are independent risk factors for various pathologies (Aizawa et al., 2021; Behnam et al., 2019; Davies et al., 2014; Hametner et al., 2014; Huang et al., 2017; Narayan et al., 2015). Correlation does not prove causality, but it does stimulate further studies. The association between reservoir pressure and the elastic energy stored in the arterial walls revealed in the above analysis should help such studies find the mechanistic links involved.

The demonstration that reservoir pressure is proportional to the elastic energy stored in the walls of the elastic arteries identifies it with the Windkessel effect first described by Stephen Hales (Hales, 1733) and later quantified by Otto Frank (Frank, 1899). This is the process which transforms the highly pulsatile nature of blood ejected by the heart into the aortic root into relatively smooth, continuous perfusion through the capillaries. The stiffness of the arteries directly affects the reservoir pressure. It seems evident, therefore, that better understanding of the reservoir pressure will be valuable in the identification and treatment of many cardiovascular pathologies, including hypertension.

The excess pressure, defined as the difference between the measured pressure and the reservoir pressure, is more difficult to describe physically because it has many possible origins. Unlike the reservoir pressure, which is uniform throughout the inviscid arteries, the excess pressure varies locally and must be associated with local variations in the flow. These variations are not necessarily pathological and could be generated by normal variations in local anatomy and the distribution of compliance. For example, excess pressure could be caused by flow separation at a bifurcation (which has been observed in normal renal arteries during early systole) or local variations in arterial compliance, which can cause local changes in excess pressure in normal subjects. Many pathologies, particularly stenoses, could affect the excess pressure, making it a non‐specific indicator. Excess pressure, however, may be a more sensitive parameter in the detection of any pathological local changes. This is supported by some studies that show that some measure of excess pressure predicts future cardiovascular disease (Davies et al., 2014; Huang et al., 2017).

A further benefit of knowing the excess pressure comes from experimental measurements made in open‐chest dogs (Tyberg et al., 2009). In this study, it was shown that the excess pressure measured in the ascending aorta was proportional to the measured velocity of blood into the root of the aorta. Figure 3 shows the excess pressure *P*_e_ calculated using measured *P* and *U* using the Windkessel equation together with the measured volume flow rate *Q* simultaneously measured at the root of the aorta. The axes of the variables are adjusted so that their peak values coincide. The shape of the two curves is remarkably similar for measured physiological data, particularly during systole. This is partly due to the dog being paced and ventilated and because some smoothing was done during the calculation of *P*_r_ from the *P* and *U* measurements. Broadly similar observations have been made in humans (Armstrong et al., 2021; Hughes et al., 2020; Vermeersch et al., 2009).

**FIGURE 3:**
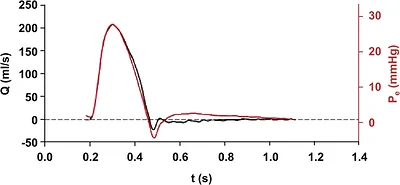
The volume flow rate Q measured at the aortic root (red) and the excess pressure $P_{e}$ measured in the ascending aorta of an open‐chest dog. The scales have been adjusted so that the maxima of the two waveforms coincide to show the similarity between the two waveforms. t=0 indicates the peak of the R‐wave in the simultaneously measured ECG.

The assumption that excess pressure is proportional to flow rate into the aortic root has been used to develop an algorithm for the calculation of reservoir pressure from the measured pressure alone (https://kparker.bg‐research.cc.ic.ac.uk//res_press_web/rp_download.html), and updated versions of this have been used in MATLAB software (MathWorks, Natick, MA, USA) for processing data recorded using SphygmoCor (https://github.com/adh30/Sphygmocor‐Reservoir; https://doi.org/10.5281/zenodo.4593774) and BP+ (https://github.com/adh30/BPplus‐Reservoir; https://doi.org/10.5281/zenodo.22228884) devices. This approach has been used in most of the studies exploring the utility of reservoir and excess pressure as risk factors.

Figure 4 shows another potential use of $P_{r}$ in cardiology. It shows the P−V and $P_{r}-V$ curves measured in the ascending aorta of an open‐chest dog and how they vary following the administration of nitroprusside and methoxamine based on data in Wang et al. (2013). The change in volume ΔV of the left ventricle is calculated from the volume flow rate Q measured at the aortic root as $\Delta V=-\int_{0}^{t}Qdt$, which follows from the conservation of mass. From its definition V(0) is the volume of the left ventricle at the end of diastole/start of systole (i.e. the maximum volume of the ventricle). The dashed vertical lines represent the volume of the ventricle at the end of systole (i.e. the minimum volume of the ventricle). The hydraulic work W=∫PdV is the integral under the curves. Thus, the total hydraulic work done by the left ventricle over one cardiac cycle is given by the area under the black curves, and the area under the red curves gives the fraction of that work contributed by the reservoir pressure *P*_r_. We can define the excess work (i.e. the work contributed by the excess pressure $P_{e}$ as the area between the black and red curves.

**FIGURE 4:**
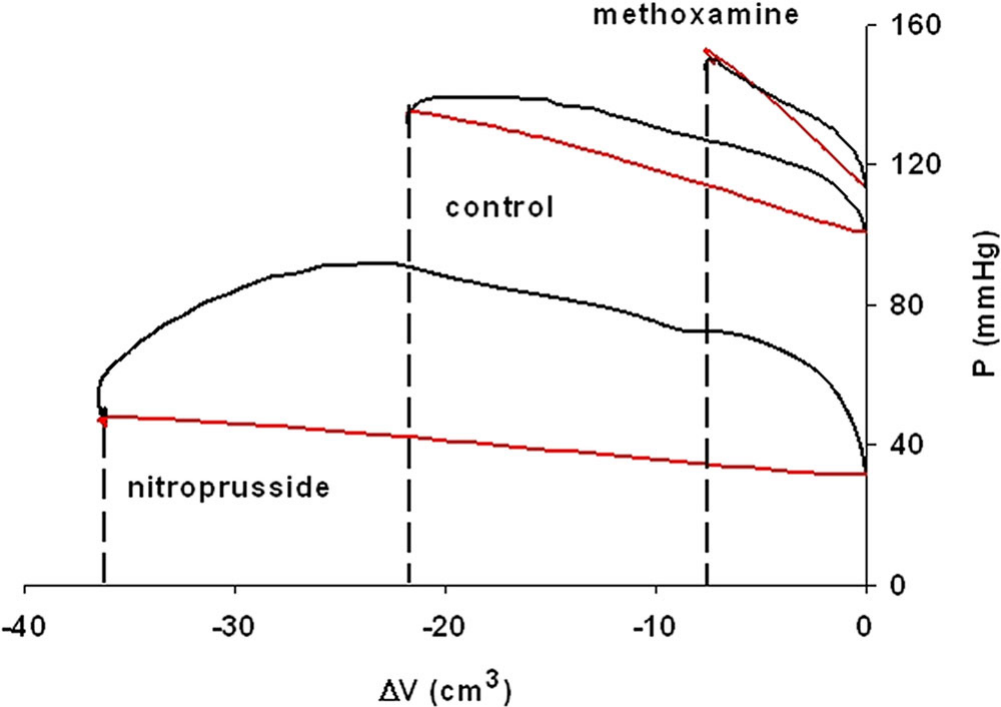
The P−ΔV (black) and $P_{r}-\Delta V$ (red) curves measured in the ascending aorta of an open‐chest dog at: (1) control conditions (middle), (2) after administration of nitroprussside (lower), and (3) after administration of methoxamine (upper). $\Delta V=-\int_{0}^{t}Qdt$, where t=0 is the end of diastole/start of systole. The area under these curves is the hydraulic work done by the left ventricle during systole.

The effect of vasoactive drugs on the excess work done by the left ventricle of this dog is dramatic. After the administration of nitroprusside, a nitrovasodilator, the excess hydraulic work is an order of magnitude larger than the control value. After administration of methoxamine, an α_1_‐adrenoceptor agonist that acts as a vasoconstrictor, the excess hydraulic work is more than an order of magnitude smaller than the control value – very nearly zero. The drug doses used in this experiment were rather large, but are illustrative of potential magnitudes of change under (patho)physiological conditions.

From these observations, we believe that the reservoir‐excess pressure hypothesis could lead to a more intuitive understanding of conditions with abnormal haemodynamics, including essential hypertension. Current US guidelines for hypertension list four classes of therapeutic agents recommended for initial therapy and a further 15 classes of alternative agents, and most people with hypertension require multiple agents to achieve BP control, typically requiring careful initiation and monitoring (Jones et al., 2025). It would be interesting to assess whether use of reservoir and excess pressure could assist with treatment selection.

The separation of arterial system into effectively inviscid and viscid arteries through the non‐dimensional drag coefficient could contribute to future models of the arteries. A direct analogy is boundary layer theory, one of the most successful techniques in applied fluid mechanics. This method involves identifying regions in the flow where viscosity is negligible (e.g. the air far from the surface of a wing) and regions where viscosity is important (e.g. the boundary layer next to the wing). The solutions in the two regions are found separately, and the full solution is found by matching the two solutions on the boundary between the regions. In most cases, the analysis is carried out using asymptotic analysis similar to that used in the theoretical derivations in this work. It is impossible to predict how important this model will be in future studies of arterial dynamics, but it holds promise.

## 4 DISCUSSION AND CONCLUSIONS

Two‐time asymptotic analysis provides a framework for analysing arterial haemodynamics. It assumes that time t can be separated into a fast‐time τ, dependent upon a characteristic wave speed $c^{*}$, and a slow‐time T, determined by the characteristic velocity of the blood $U^{*}$. The ratio of these characteristic times is the Mach number $m=U^{*}/c^{*}$, which is generally small for arterial flows. In some young individuals m may not be as small, since arterial wave speeds are lower, which suggests that the reservoir hypothesis should be used with due caution in the young (Kodithuwakku et al., 2025).

Applying the two‐time analysis to the 1‐D conservation equations for mass and momentum shows that to O(m) the solution for the pressure and velocity obeys the wave Equation (12) on the fast‐time scale and simple relationships involving the convective derivative on the slow‐time scale. The wave equation is hyperbolic, and solutions depend only on the boundary conditions at the inlet and outlets of the arterial system and matching conditions at discontinuities such as bifurcations within the arterial network. The fast‐time solutions depend on the boundary conditions at the slow‐time scale.

The slow‐time conservation equations involve a resistance term (the viscous drag coefficient R) and leads to the suggestion that the arteries can be subdivided into inviscid arteries where viscous effects can be neglected and viscid arteries where viscous losses are important. This assumption is similar in principle to the boundary layer approximation in aerodynamics. In the inviscid arteries the slow‐time conservation equations and the Kirchoff conditions at the bifurcations show that the pressure is homogeneous in space and variable in time. We identify this pressure with the reservoir pressure $P_{r}$ originally introduced in the heuristic reservoir‐excess hypothesis.

We apply overall mass conservation to the inviscid arteries to calculate the dynamics of $P_{r}$ as a function of the known volume flow rate into the aortic root from the left ventricle $Q_{0}\left(t\right)$, the net resistance of the viscid arteries and the microcirculation R and the net compliance of the inviscid arteries C.

The physical meaning of the reservoir pressure, revealed by its theoretical derivation, is straightforward: it is the potential energy stored by the elastic walls of the inviscid arteries during the cardiac cycle. The energy stored during the early part of systole is recovered during late systole and particularly during diastole when it generates flow through the viscid arteries and microcirculation; this smooths out the highly pulsatile flow generated by the ventricle. This corresponds to the notion of the ‘arterial reservoir’ held by many physiologists and cardiologists and explains why the Windkessel has remained a useful teaching concept. This interpretation is also supported by an earlier analysis using the calculus of variation to show that the hydraulic work done by the reservoir pressure is the minimum work necessary to generate any given flow rate in the arteries (Parker, 2013).

Some researchers (Mynard & Smolich, 2014; Westerhof et al., 2015) have criticised the reservoir‐excess pressure hypothesis. We believe these criticisms were mainly a result of a misunderstanding about the role of waves in generating the reservoir pressure. Both slow and fast time components are due to waves but viewed on different time scales. The two‐time analysis that is the basis of our theoretical approach should clarify these misunderstandings and lead to a wider acceptance of reservoir pressure as a useful concept in arterial haemodynamics.

Relatedly, it has been proposed that $P_{r}$ is directly related to the backward wave pressure $P_{b}$ that is obtained when the measured P is separated into its forward and backward components (Westerhof et al., 2015). It is true that $P_{r}\approx 2P_{b}$ during diastole when Q=0 (and hence $P_{f}=P_{b}$), but it is not true during systole when Q≠0, and $P_{r}$ calculated using the methods discussed above may be different from $2P_{b}$. The difference between *P*_r_ and 2*P*_b_ can be seen in Figure 5 where the forward *P*_f_ and backward *P*_b_ pressures due to the forward and backward waves (dashed lines) are calculated using wave intensity analysis and the wave speed used in the numerical calculations.

**FIGURE 5:**
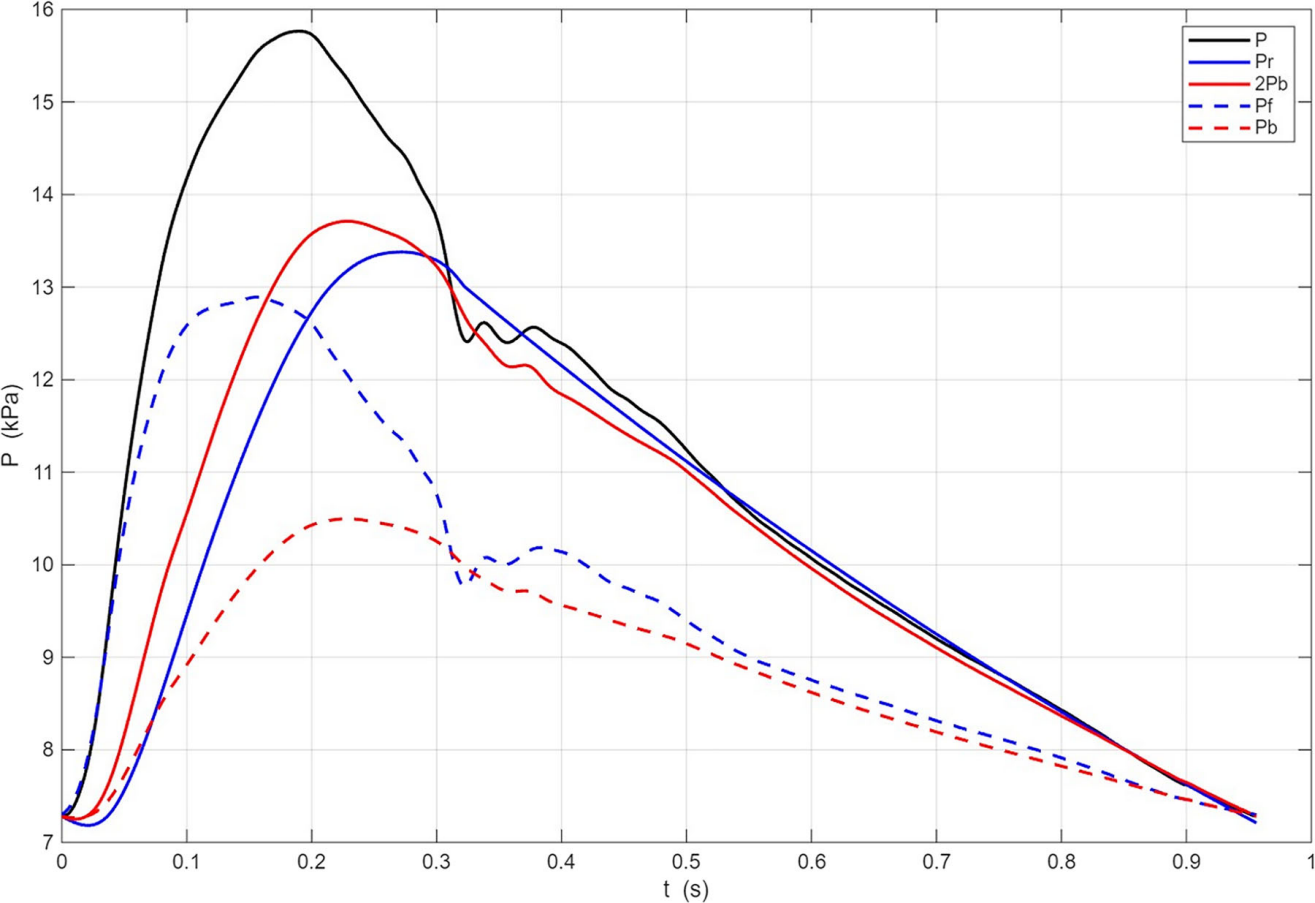
Various pressures calculated in the human brachial artery. The pressure *P* (black line) is the same as in Figure 1. The forward (blue dashed line) and backward (red dashed line) pressures are calculated using wave intensity analysis and the wave speed used in the numerical computation. Two times the backward pressure (red line) is also shown because it has been suggested that this is an alternative way to calculate reservoir pressure. The difference is significant during systole and smaller, but noticeable, during diastole.

Another misunderstanding about reservoir pressure comes from 1‐D computational studies of arterial models where the first waves returning to the aortic root deviate from the reservoir pressure (Mynard & Smolich, 2014). The two‐time analysis shows that the reservoir pressure results from the boundary conditions established by all of the waves in the arterial system, not just the early reflected waves. In fact, it is likely that some of the wavelets determining the reservoir pressure are very old, having been generated by ventricular contractions several beats before the current beat (Willemet & Alastruey, 2015). These wavelets are very small in intensity due to the large number of reflections and re‐reflections they have experienced, but there is an enormous number of them, and it is not obvious that they can be neglected. It is clear, however, that $P_{r}$ is not the resultant of the first few reflections that are calculated by computational modelling.

This study gives the reservoir pressure a rigorous theoretical basis for the first time. The two‐time analysis is an approximate asymptotic analysis, but it is mathematically rigorous and gives us a quantitative bound on the approximations that have been made; by definition, the O(m) results are accurate to $O(m^{2})$, where m is the characteristic Mach number for arterial flows. Since arterial wave speeds are typically at least an order of magnitude greater than the mean velocity, the results should be accurate to within a few percent. This is commensurate with the accuracy of other studies of biological flows – experimental and theoretical.

To make these arguments more quantitative, we show some estimates of the two asymptotic parameters m and r for a range of arteries in Table 1. All of the values in the table are approximate because of the large variation of cardiovascular parameters between subjects and at different states within a subject. The basic data have been selected by the authors from a wide range of sources including answers provided by various AI platforms (although the accuracy of the latter has been checked by the authors).

**TABLE 1:** Estimates of physiological variables under resting conditions for a range of arteries.

Artery	*U*	*c*	*m*	*D*	*L*	Re_D_	*r*
	(m/s)	(m/s)		(m)	(m)		
Torso							
Ascending aorta	0.45	4.3	0.11	0.029	0.040	3400	0.013
Aortic arch	0.74	4.3	0.17	0.022	0.059	4300	0.020
Thoracic aorta	0.74	4.7	0.16	0.015	0.052	2900	0.038
Renal	0.50	7.5	0.067	0.0060	0.050	790	0.34
Abdominal aorta	0.30	4.8	0.063	0.012	0.18	950	0.50
Superior mesenteric	0.40	6.0	0.067	0.0075	0.03	790	0.16

Head							
Common carotid	0.35	5.3	0.066	0.0074	0.18	680	1.1
Internal carotid	0.40	4.8	0.083	0.0048	0.085	500	1.1
Retinal	0.098	10	0.010	0.00016	0.020	4	970

Arm							
Brachial	0.50	5.9	0.085	0.0064	0.42	840	2.5
Radial	0.80	9.5	0.084	0.0032	0.24	670	3.5
Digital	0.06	10	0.006	0.0010	0.039	21	96

Leg							
Iliac	0.55	7.7	0.071	0.0090	0.060	870	0.89
Femoral	0.14	9.5	0.015	0.0070	0.040	260	0.71
Anterior tibial	0.14	8.6	0.016	0.0044	0.44	160	20

Because of the wide range of these variables between individuals and within individuals under different physiological conditions, the estimates are rounded to two significant figures. All dimensional parameters are given in SI units.

Abbreviations: *c*, wave speed; *L*, length; *m*, Mach number; *r*, non‐dimensional viscous resistance parameter; Re_D_, Reynold number; *U*, flow velocity.

Looking first at the Mach number m, we see that the two‐time asymptotic analysis is credible. The largest m=0.17 is in the thoracic aorta, which means the results should be within approximately 3% of the true (and unobtainable) theoretical results. The accuracy of the analysis in the other arteries is much better, and we expect that the results of the asymptotic analysis can be used with confidence in physiology and medicine.

The second important innovation in this analysis, the separation of the arteries into inviscid and viscid arteries, depends on the dimensionless drag coefficient r. The critical value of r in this categorisation is not immediately obvious and probably depends upon the particular problem being considered. In engineering fluid mechanics, boundary layer methods are frequently used for Reynolds numbers Re in the hundreds. From the table we see that the proximal aorta can certainly be treated as an inviscid artery. If we take r<5 as a rough threshold, then the inviscid arteries would include the whole aorta and the large conduit arteries in the arms and legs and, presumably, the large abdominal systemic arteries that are not included in the table.

Many previous studies of haemodynamics in the large arteries have ignored viscosity, but they have done it implicitly. Our approach has the advantage that it quantifies the level of approximation that is being made. This categorisation of the systemic arteries into inviscid and viscid arteries has been useful in defining the extent of the utility of reservoir pressure. The extension of this idea to the analysis of the viscid arteries may shed some welcome light on the physiology of the small arteries, an important and grey area in our understanding of the fluid dynamics of the cardiovascular system. We note that *r* <5 (with some assumptions) would correspond to small muscular arteries and arterioles with internal diameter ≤3 mm; this is roughly in line with a physiological definition of muscular and resistance arteries (Christensen & Mulvany, 2001).

Finally, the most important result of this analysis for future studies is that haemodynamics in the arteries can be considered to be quasi‐steady to O(m); that is, terms of magnitude $m^{2}$ have been ignored. Physically, this is because the wave speed is large enough to establish steady‐state conditions at every interval on the slow time scale of variations in the boundary conditions that determine the fast time scale wave solutions. This is an approximate result made quantitative by the two‐time asymptotic analysis we have used. The quasi‐steady solutions are not easy to calculate because of the size and anatomical complexity of the arterial tree. They are, however, substantially simpler than the full solutions of the underlying partial differential conservation equations, which challenge even numerical solutions using computational fluid dynamics (CFD).

Current 3‐D CFD solutions are generally limited to two or three bifurcations with highly simplified boundary conditions. 1‐D CFD can calculate mean flow and pressure conditions on networks of up to 100–200 arteries and is very useful (Xiao et al., 2014). Extending these 1‐D simulations is limited less by the size of the network than by the lack of knowledge about the anatomy and properties of the smaller arteries. Given the estimate that there are approximately 2 billion arteries in the human body, it is obvious that the numerical result for a few hundred arteries is literally scratching the surface of the problem. We believe that progress in theoretical haemodynamics must involve more intelligent modelling of the small arteries, which can lead to a better understanding of physiologically important parameters. This can, in turn, lead to better experiments to determine experimental measurement of them, possibly involving theoretically informed averaging of imaging data from the many available modalities.

Being theoretically rigorous, of course, does not ensure that the results of the analysis are useful. (The most memorable expression of this principle comes from Yogi Berra (1925–2015), who played catcher for the New York Yankees baseball team: ‘In theory there is no difference between practice and theory, in practice there is.’ [WikiQuotes]) That question can only be answered by clinical studies and practice. However, the theoretical results give us confidence in the validity of the concept of separating measured arterial pressure into reservoir pressure and excess pressure. It also gives us insight into the physical basis of reservoir pressure, which may be useful in understanding future clinical results.

## AUTHOR CONTRIBUTIONS

Kim H. Parker developed the theoretical formulation and took the lead in drafting the manuscript. Alun D. Hughes contributed some ideas and revised the manuscript. Both authors discussed the contents and interpretation and contributed to the final manuscript. Both authors have read and approved the final version of this manuscript and agree to be accountable for all aspects of the work in ensuring that questions related to the accuracy or integrity of any part of the work are appropriately investigated and resolved. All persons designated as authors qualify for authorship, and all those who qualify for authorship are listed.

## CONFLICT OF INTEREST

None declared.

## GENERATIVE AI STATEMENT

Some data included in Table 1 were identified using a range of AI platforms. The accuracy of the data was checked by the authors.

## Data Availability

The data that support the findings of this study are available from the corresponding author upon reasonable request.
